# CHA2DS2-VASC score as a preprocedural predictor of contrast-induced nephropathy among patients with chronic total occlusion undergoing percutaneous coronary intervention: a single-center experience

**DOI:** 10.1186/s12872-019-1060-0

**Published:** 2019-03-29

**Authors:** Yong Wang, Hong-wei Zhao, Xiao-jiao Zhang, Bao-jun Chen, Guo-ning Yu, Ai-jie Hou, Bo Luan

**Affiliations:** 1 Department of Cardiology, The Peple’s Hospital of China Medical University, The Peple’s Hospital of Liaoning Province, No. 33, Wenyi road, Shenhe District, Shenyang City, 110016 Liaoning Province China; 2 Department of Science and Education, The Peple’s Hospital of China Medical University, The Peple’s Hospital of Liaoning Province, Shenyang, China

**Keywords:** Chronic total occlusion, CHA2DS2-VASC score, Percutaneous coronary intervention, Contrast-induced nephropathy

## Abstract

**Background:**

The usefulness of the CHA2DS2-VASC risk score (CVRS) in predicting the occurrence of contrast-induced nephropathy (CIN) among patients with chronic total occlusion (CTO) undergoing percutaneous coronary intervention (PCI) remains unclear.

**Method:**

A total of 239 patients with CTO who underwent PCI were included in this study. They were divided into 3 groups according to the CVRS: low-risk group (1 point, *n* = 64), intermediate-risk group (2 points, *n* = 135), and high-risk group (≥3 points, *n* = 40). Baseline serum creatinine was determined upon admission before the procedure. The serum creatinine level was monitored for 72 h post-procedure to determine the occurrence of CIN.

**Results:**

The total incidence of CIN in patients with CTO who underwent PCI was 16.3%. The average CVRS in the CIN group was significantly higher than that in the non-CIN group (3.1 ± 1.2 VS 2.1 ± 1.1, *P* < 0.001). The incidence of CIN in the high-risk group was 5.6 times higher than that in the low-risk group (37.5% VS 6.3%, *P* < 0.001). Similar to the Mehran risk score (AUC, 0.754; 95% CI, 0.698–0.810; P < 0.001), the receiver operating characteristic curve analysis showed a good diagnostic value of the CVRS in predicting CIN among patients with CTO who underwent interventional therapy for having CVRS≥3 (sensitivity, 69.2%; specificity, 78.0%; AUC, 0.742; 95% CI, 0.682–0.797; *P* < 0.001). The multivariate analysis showed that the higher pulse pressure and contrast volume, lower baseline glomerular filtration rate, and CVRS ≥3 were independent predictors of CIN.

**Conclusions:**

The CVRS can be used as a simple pre-procedural predictor of CIN among patients with CTO undergoing PCI.

## Highlight of this research

1. CHA2DS2-VASC score was an predictor of CIN for patients with CTO undergoing PCI.
2. It can be used to Identify high risk patients and prepare therapeutic intervention.
3. It has a similar predictive value to Mehran risk score.
4. CHA2DS2-VASC scoring may be convenient and easily applied in clinical practice.

## Background

The CHA2DS2-VASC risk score (CVRS) was developed for embolic risk stratification in patients with atrial fibrillation (AF) to provide further optimal anticoagulant therapy [1]. Studies have confirmed that the CVRS could be used for the prediction of coronary artery disease [2, 3] and long-term prognosis in patients undergoing percutaneous coronary intervention (PCI) [4, 5]. Moreover, it was feasible in predicting acute stent thrombosis in AF-free patients [6] and the no-reflow phenomenon among patients with ST-segment elevation myocardial infarction (STEMI) who underwent primary PCI [7]. Since the CVRS is widely used, whether it can be useful to predict contrast-induced nephropathy (CIN), which is one of the most common complications in patients who undergo PCI, is unclear. Evidences have suggested that the scoring system also has a predictive value for CIN after PCI among patients with acute coronary syndrome (ACS) [8] and STEMI [9]. However, the usefulness of the CVRS in predicting the occurrence of CIN among patients with chronic total occlusion (CTO) undergoing PCI remains unknown. In this study, we aimed to determine CVRS as a predictor of CIN among these patients.

## Method

### Study population

From January 2016 to November 2017, we consecutively admitted 239 patients with CTO lesions diagnosed by coronary angiography (CAG) in our hospital. Baseline serum creatinine was determined upon admission. The serum creatinine level was monitored for 72 h after the procedure to determine the occurrence of CIN. Exclusion criteria included patients who underwent haemodialysis or those with glomerular filtration rate (GFR) < 15 mL/min/1.73 m^2^, severe heart failure [New York Heart Association (NYHA) IV], pulmonary oedema, recent (past 2 days) use of contrast, and the use of potential nephrotoxic drugs within 72 h prior to the procedure and 72 h after the catheterization. PCI was performed among patients with angina or silent ischaemia with viable myocardium in the occluded coronary artery using the myocardial nuclear scan, stress dobutamine echocardiography, or cardiac magnetic resonance imaging.

All patients were prescribed a loading dose of aspirin 300 mg and clopidogrel 300 mg prior to the procedure. The CAG was performed via the radial artery approach, and bilateral CAG was performed when necessary. We attempted to open the CTO lesion using antegrade crossing techniques. The femoral artery path was used during vasospasm or vascular tortuosity or based on the operator’s decision. Retrograde crossing techniques were used if the antegrade crossing techniques failed and the patient had a good collateral circulation. Heparin 100 U/kg was administered as an anticoagulant. The use of glycoprotein IIb/IIIa receptor inhibitor and the type of stents were based on the physician’s discretion. All patients signed an informed consent.

Iopromide [for patients with estimated GFR (eGFR) ≥40 mL/min/1.73 m^2^] and iodixanol (for patients with eGFR < 40 mL/min/1.73 m^2^) were used during the procedure. Patients with a baseline eGFR < 40 mL/min/1.73 m^2^ received intravenous hydration with a standard normal saline at a rate of 1 mL/kg/h (or 0.5 mL/kg/h in patients with heart failure) for at least 12 h before and after the cardiac catheterization. Potential nephrotoxic drugs were withdrawn for at least 72 h before and after the catheterization.

### Definitions

CTO is defined as an obstruction of the coronary arteries with Thrombolysis In Myocardial Infarction (TIMI) flow grade 0 with an estimated duration of at least 3 months [1, 10]. CIN was defined as a creatinine increase of at least 0.5 mg/dL or ≥ 25% from the baseline within 72 h following cardiac catheterization [11]. The Cockroft-Gault formula and modification of diet in renal disease equation were used to determine the baseline eGFR [12, 13]. Severe renal dysfunction (SRD) was defined as acute renal failure requiring dialysis or at least 2.0 mg/dL or ≥ 50% of creatinine elevation from the baseline within 24 h after the procedure [14]. Angiographic success was defined as residual stenosis ≤30% by visual analysis in the presence of TIMI flow grade 2–3.

### Study end points

The primary end point was the occurrence of CIN whereas the secondary end point was severe renal dysfunction requiring haemodialysis.

### Statistical analysis

Normally distributed continuous variables were expressed as mean ± standard deviation, and non-normally distributed variables were represented as median (min-max). Similarly, categorical variables were expressed as percentages. To compare the differences of continuous data, the analysis of variance was used to analyse parametric data, and the Kruskal–Wallis H test was carried out for nonparametric data. Categorical data were analysed using the Chi-square or Fisher’s exact test based on the actual situation. The receiver operating characteristic (ROC) curve analysis was used to determine the optimum cutoff values of the CVRS to predict the incidence of CIN. Additionally, the logistic regression model was used to determine the independent predictors of CIN that were not included in the CVRS. A *P*-value < 0.05 was considered statistically significant.

## Result

### Lesion and procedural characteristics

A total of 239 patients with CTO (82 females, 34.3%) who underwent angiography were included in this study, and all enrolled patients were followed-up for 72 h after the procedure. The incidence of CIN was 16.3%. In this study, the incidence of CTO lesions was predominant in the right coronary artery (97, 40.6%). Transradial approach was the predominant access route (69%). The retrograde approach accounted for 23.8% of the procedures, and the success rate of the operation was 92.1%. None of the patients had SRD which required early dialysis and major bleeding which needed transfusion; however, a groin haematoma > 5 cm was observed in 2.1% (*n* = 5) of the patients.

### Comparison among the low-risk, intermediate-risk, and high-risk groups

The mean age of our study population was 59.4 ± 9.9 years, and the mean CVRS was 2.3 ± 1.3. The patients’ demographic and clinical characteristics were compared among the 3 groups (Table 1). Data on the age, female gender, and the incidence of hypertension, pulse pressure, diabetes mellitus, stroke, and NYHA II–III on admission were higher in the group with CVRS ≥4. The patients in the high-risk group had higher pulse pressure, total contrast volume, total procedure time, rate of intra-aortic balloon pump (IABP) insertion, and number of stent implantation and lower eGFR and diastolic blood pressure. The overall rate of CIN was 16.3%, and a significant difference was noted in the high-risk group compared to the low-risk and intermediate-risk groups (6.3% VS 14.8% VS 37.5%, *P* < 0.001).

**Table 1:** Clinical characteristics of study population according to CHA2DS2-VASC

Variable	CHA2DS2-VASc Score			*p*-value
	low risk (1 point, *n* = 64)	intermediate risk (2–3 points, *n* = 135)	high risk (≥4 points, *n* = 40)	
Age (years), mean (SD)	53.0 ± 7.5	59.1 ± 6.4	67.9 ± 7.9	**P<0.001**
Gender (female), n(%)	0	63 (47.4)	19 (47.5)	**P<0.001**
Body mass index (Kg/m^2^)	25.3 ± 1.8	24.4 ± 2.9	24.3 ± 2.6	**0.04**
Diabetes Mellitus, n(%)	0	20 (14.8)	20 (50.0)	**P<0.001**
Hypertension, n(%)	0	34 (25.2)	27 (67.5)	**P<0.001**
Stroke history, n(%)	0	2 (1.5)	6 (15.0)	**P<0.001**
Current smoker, n(%)	17 (26.6)	45 (33.3)	8 (20.0)	0.23
Previous MI, n(%)	19 (29.2)	46 (34.1)	11 (25.5)	0.67
Systolic blood pressure (mmHg)	119.1 ± 13.7	121.8 ± 12.1	124.6 ± 14.2	0.28
Diastolic blood pressure (mmHg)	74.7 ± 10.0	74.2 ± 9.3	72.4 ± 5.8	**0.015**
Pulse pressure (mmHg)	44.4 ± 10.2	47.6 ± 9.7	52.2 ± 12.2	**0.001**
LVEF	0.51 ± 0.04	0.45 ± 0.06	0.43 ± 0.04	**P<0.01**
NYHA 2–3 on admision	0	20 (14.8)	17 (42.5)	**P<0.01**
Total Cholesterol (mmol/L)	4.2 ± 1.0	4.3 ± 1.1	4.3 ± 0.9	0.70
LDL-C (mmol/L)	2.6 ± 0.6	2.5 ± 0.6	2.7 ± 0.6	0.23
HDL-C (mmol/L)	1.1 ± 0.3	1.1 ± 0.3	1.2 ± 0.4	0.25
Triglyceride (mmol/L)	1.6 ± 1.2	1.5 ± 1.3	1.3 ± 0.8	0.59
Fasting Glucose (mmol/L)	5.3 ± 0.5	5.5 ± 0.8	5.8 ± 1.0	**0.027**
eGFR baseline (ml/min/1.73m^2^)	102.0 ± 13.8	92.8 ± 17.0	89.5 ± 17.6	**P<0.001**
eGFR after PCI (ml/min/1.73m^2^)	98.4 ± 14.2	87.4 ± 19.5	76.2 ± 21.3	**P<0.001**
First Day Creatinine (μmol/l)	68.8 ± 19.2	69.5 ± 16.9	65.0 ± 17.6	0.37
Uric acid (μmol/l)	330.3 ± 69.9	330.8 ± 69.8	336.1 ± 75.6	0.90
Total amount of conrrast (ml)	181.8 ± 63.5	241.8 ± 104.0	320.3 ± 92.5	**P<0.001**
Total time of procedure (min)	74.4 ± 45.6	96.1 ± 47.7	129.7 ± 51.6	**P<0.001**
The retrograde approach, n(%)	14 (21.5)	29 (21.5)	14 (35.0)	0.19
Transradial + transfemoral approach, n(%)	21 (32.8)	42 (31.1)	17 (42.5)	0.40
IABP, n(%)	4 (6.3)	6 (4.4)	7 (17.5)	**0.02**
IVUS, n(%)	4 (6.3)	9 (6.7)	5 (12.5)	0.42
Stent number	1.9 ± 0.3	2.3 ± 0.6	2.6 ± 1.1	**P<0.001**
Glycoprotein IIb/IIIa receptor inhibitor, n(%)	12 (18.8)	24 (17.8)	13 (32.5)	0.12
CIN	4 (6.3)	20 (14.8)	15 (37.5)	**P<0.001**

*MI* myocardial infarction, *LVEF* left ventricular ejection fraction, *NYHA* New York Heart Association (classification), *LDL-C* low density lipoprotein-cholesterol, *HDL-C* high density lipoprotein-cholesterol, *IABP* intra-aortic balloon pump, *IVUS* intravascular ultrasound, *CIN* contrast induced nephropathy

### Comparison between the CIN and non-CIN group

**Fig. 1:**
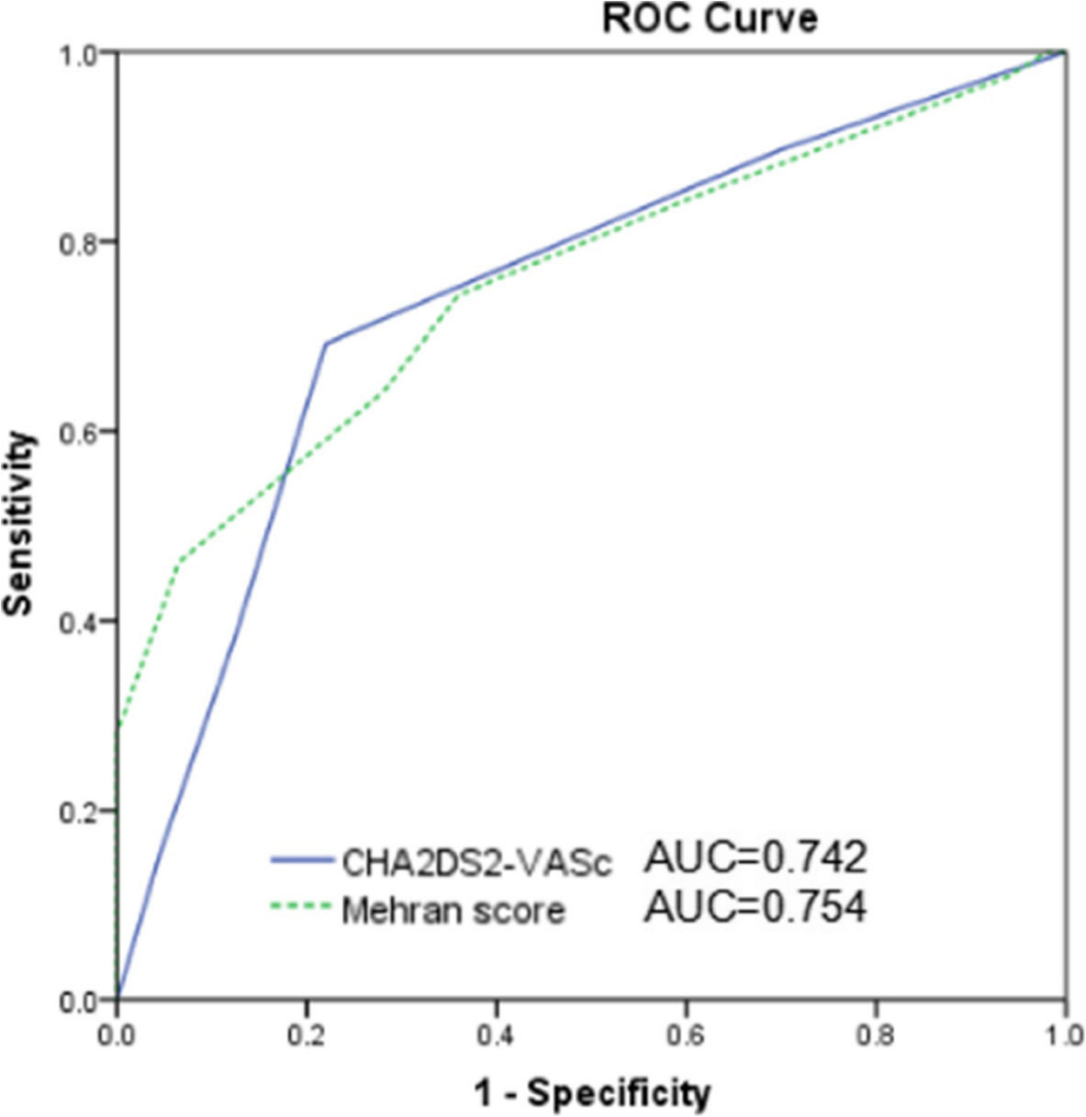
Receive-operating characteristic (ROC) curve analysis for presence and number of CHA2DS2-VASC scores for predicting contrast-induced nephropathy

The incidence of CIN was 16.3%. Table 2 demonstrates that patients diagnosed with CIN were older and required longer procedure time. A significant difference was observed in the age, female, systolic and diastolic blood pressure, pulse pressure, and incidence of diabetes mellitus, hypertension, and stroke history between the 2 groups. Furthermore, patients with CIN had higher LDL-C, fasting glucose, uric acid, total contrast volume, rate of glycoprotein IIb/IIIa receptor inhibitor, and CVRS than those without CIN (3.1 ± 1.2 VS 2.1 ± 1.1; *P* < 0.001). The ROC curve analysis revealed that the area under the curve for predicting CIN was 0.742 (sensitivity, 69.2%; specificity, 78.0%; 95% CI, 0.682–0.797; P < 0.001) for CVRS ≥3 (Fig. 1). The incidence of CIN increased as the risk score increased. Multivariate analysis showed that higher pulse pressure [odds ratio (OR), 1.042; 95% CI, 1.012–1.197; *P* = 0.004] and contrast volume (OR, 1.772; 95% CI, 1.342–2.128; *P* = 0.039), lower baseline eGFR (OR, 0.662; 95% CI, 0.521–0.789; *P* = 0.012), and CVRS ≥3 (OR, 6.679; 95% CI, 3.169–15.531; *P* < 0.001) were independent predictors of CIN pre-procedure in CTO patients (Table 3).

**Table 2:** Clinical characteristics of the patients with and without contrast-induced nephropathy

Variable	contrast-induced nephropathy		*P*-value
	Yes (*n* = 39)	NO (*n* = 200)	
Age (years), mean (SD)	58.4 ± 8.4	64.5 ± 14.7	**P<0.001**
Gender (female), n(%)	17 (43.6)	65 (32.5)	0.13
Body mass index (Kg/m^2^)	24.6 ± 2.7	24.7 ± 2.4	0.85
Diabetes Mellitus, n(%)	11 (28.2)	29 (14.5)	**0.04**
Hypertension, n(%)	21 (53.8)	40 (20.0)	**P<0.001**
Stroke history, n(%)	5 (12.8)	3 (1.5)	**0.004**
Current smoker, n(%)	7 (17.9)	63 (31.5)	0.06
Previous MI, n(%)	15 (38.5)	50 (25.0)	0.11
Systolic blood pressure (mmHg)	120.6 ± 12.6	126.5 ± 13.8	**0.009**
Diastolic blood pressure (mmHg)	74.5 ± 9.2	72.1 ± 8.1	**P<0.001**
Pulse pressure (mmHg)	54.4 ± 12.1	46.1 ± 9.7	**P<0.001**
LVEF	0.47 ± 0.07	0.44 ± 0.06	**0.02**
NYHA 2–3 on admision	7 (17.9)	30 (15.0)	0.40
Total Cholesterol (mg/dl)	4.4 ± 0.7	4.3 ± 1.1	0.33
LDL-C (mmol/L)	2.8 ± 0.5	2.5 ± 0.6	**0.007**
HDL-C (mmol/L)	1.0 ± 0.2	1.1 ± 0.3	0.09
Triglyceride (mmol/L)	1.4 ± 0.6	1.5 ± 1.3	0.35
Fasting Glucose (mmol/L)	5.4 ± 0.7	5.8 ± 1.3	**0.004**
Baseline eGFR (ml/min/1.73m^2^)	94.6 ± 17.6	92.7 ± 20.3	0.53
Baseline Creatinine (μmol/l)	69.2 ± 18.0	65.3 ± 15.6	0.21
Uric acid (μmol/l)	355.4 ± 72.4	326.9 ± 69.4	**0.02**
Total amount of conrrast (ml)	299.2 ± 105.2	227.1 ± 98.3	**P<0.001**
The retrograde approach, n(%)	6 (15.4)	51 (25.5)	0.12
Transradial + transfemoral approach, n(%)	12 (30.8)	68 (34.0)	0.85
Procedural duration (min)	91.0 ± 50.0	120.9 ± 48.4	**P<0.001**
IABP, n(%)	3 (7.7)	14 (7.0)	0.75
IVUS, n(%)	4 (10.3)	14 (7.0)	0.51
Stent number	2.2 ± 0.9	2.2 ± 0.6	0.96
Glycoprotein IIb/IIIa receptor inhibitor, n(%)	18 (46.2)	31 (15.5)	**P<0.001**
CHA2DS2-VASc Score	3.1 ± 1.2	2.1 ± 1.1	**P<0.001**

*MI* myocardial infarction, *LVEF* left ventricular ejection fraction, *NYHA* New York Heart Association (classification), *LDL-C* low density lipoprotein-cholesterol, *HDL-C* high density lipoprotein-cholesterol, *IABP* intra-aortic balloon pump, *IVUS* intravascular ultrasound

**Table 3:** Independent Predictors of Pre-procedural Contrast-Induced Nephropathy in Patients with CTO

Variable	Univariate analysis		Multivariate analysis	
	OR	*P*-value	OR(95%)	*P*-value
Pulse pressure (mmHg)	1.126	**0.042**	1.042 (1.012–1.197)	**0.014**
LDL-C (mg/dl)	1.014	**<0.001**	1.174 (1.023–1.347)	0.492
Uric acid (μmol/l)	1.008	**0.029**	1.002 (1.000–1.013)	0.193
Baseline eGFR (ml/min/1.73m^2^)	0.549	**<0.001**	0.662 (0.521–0.789)	**0.012**
Total amount of conrrast (ml)	1.971	**<0.001**	1.772 (1.342–2.128)	**0.039**
CHA2DS2-VASC risk score ≥ 3	7.743	**<0.001**	6.679 (3.169–15.531)	**<0.001**

*LDL-C* low density lipoprotein-cholesterol

## Discussion

This is the first study demonstrating that CVRS ≥3 was an independent predictor of CIN among patients with CTO who underwent PCI.

CIN is one of the most important complications of PCI, especially in patients with CTO lesions, and its pathogenesis is still not completely elucidated. It is a common complication and iatrogenic renal failure following invasive procedures, resulting in increased medical resources, longer hospital stay, and higher mortality [15–19]. According to the literature, the incidence of CIN is between 0.6 and 2.3% after contrast exposure in the general population [20]. A systematic review revealed that the incidence of CIN is approximately 3.8% among patients with CTO undergoing PCI [21]. Although identification of high-risk patients for CIN is challenging before the procedure, other studies suggested that congestive heart failure, hypertension, advanced age, diabetes mellitus, female gender, and pre-existing renal insufficiency are risk factors for CIN [22–25].

The CVRS was traditionally used for embolic risk stratification in AF patients to provide further optimal anticoagulant therapy [1]. Previous studies confirmed that CVRS could be used for the prediction of coronary artery disease [2, 3] and long-term clinical outcomes in patients undergoing PCI [4, 5]. Moreover, it was feasible in predicting acute stent thrombosis in AF-free patients [6] and the no-reflow phenomenon among patients with STEMI who underwent emergency PCI [7]. The elements of the CVRS include similar risk factors for CIN. Evidences had suggested that the scoring system also has a predictive value for CIN after PCI for patients with ACS [8] and those with STEMI who underwent emergency PCI [9]. However, individuals with CTO who underwent PCI were excluded in the study. In this study, we evaluated the CVRS and confirmed its predictive value for CIN among CTO patients who underwent PCI. Meanwhile, the results showed that the incidence of CIN was significantly higher in the high-risk group.

In this study, the CVRS had a similar predictive value with the Mehran risk score, which is the most widely used and classic model for predicting CIN. However, it is used for CIN risk assessment only after contrast medium exposure, which is restricted in clinical practise. In addition, inclusion of peri-procedural factors may restrict the application of precautionary measures before the procedure. Although CVRS excludes peri-procedural factors (e.g. contrast volume), it has a similar predictive value to the Mehran risk score. Patients with CTO undergoing PCI may be older and have poor cardiac and renal function, which are risk factors of CIN. The long procedure time for CTO-PCI requires a large contrast volume, which adds to the problem of CIN. Hence, it is of utmost clinical importance to identify high-risk patients for CIN before PCI and prepare pre-procedural therapeutic intervention to minimise the risk of such complication.

In addition, CVRS is widely used in clinical practise and it is easy to be calculated and remembered. We found that the incidence of CIN was 5.6 times higher in the high-risk group than that in low-risk patients according to the CVRS. Thus, we need to pay attention to high-risk patients and initiate preventive measures to minimise the risk of CIN, such as intravenous hydration and sodium bicarbonate and N-acetylcysteine administration before the procedure [26, 27]. Compared to other CIN risk stratification tools, the CHA2DS2-VASC scoring system may be convenient and easily applied in clinical practise.

Similar to a previous study, we discovered that higher pulse pressure level [25], which is not included in the CVRS, is an independent predictor of CIN. Perhaps an elevated pulse pressure may be transmitted to the glomerulus, and thus, impair the normal autoregulation of renal blood flow. If this persists, early renal insufficiency may occur, leading to the development of CIN [28]. Hence, these factors should be taken into consideration for predicting the incidence of CIN before PCI. Contrast volume is an important predictor of CIN; therefore, decreasing the contrast dose to reduce the incidence of CIN is helpful [29]. However, it is a predictor of CIN post-procedure, so there is a certain lag for the prediction of CIN. Further investigations are needed to confirm the results of our study.

### Limitations

This study had some limitations. First, consecutive cases of patients with CTO who underwent PCI were enrolled in the study; however, some patients were excluded because they were unsuitable for PCI or for other reasons (e.g. GFR < 15 mL/min/1.73 m^2^, severe heart failure, absence of creatinine within 72 h after the procedure), which may have led to some bias in this study. Second, this is a single-centre study; therefore, a large-scale multi-centre study is needed to further confirm these results. Finally, some risk factors of CIN, such as proteinuria, were not included in this study.

## Conclusion

The CVRS can be used as a simple pre-procedural predictor of CIN among patients with CTO who undergoing interventional therapy.

## Abbreviations

CIN: Contrast-induced nephropathy
CTO: Chronic total occlusion
CVRS: CHA2DS2-VASC risk score
eGFR: Estimated glomerular filtration rate
PCI: Percutaneous coronary intervention
TIMI: Thrombolysis in myocardial infarction
